# Modern anatomical locking plates are associated with increased postoperative wound complications and unplanned surgical revisions compared to standard tubular plates in the management of unstable ankle fractures: a comparative cohort study in 595 patients

**DOI:** 10.1186/s13037-024-00419-7

**Published:** 2024-12-03

**Authors:** Patrick Gahr, Manuel Matthis, Lennart Schleese, Dagmar-C. Fischer, Thomas Mittlmeier

**Affiliations:** 1 Department of Trauma, Hand and Reconstructive Surgery, Rostock University Medical Center, Schillingallee 35, 18057 Rostock, Germany; 2 Department of Pediatrics, Rostock University Medical Center, Rostock, Germany

**Keywords:** Fibula, Ankle fracture, Anatomical plate, Locking plate, One-third tubular plate, Mechanical complications, Wound complications

## Abstract

**Background:**

The clinical benefit of locking plates in distal fibula fractures has not yet been proven. In addition, the risk of wound complications appears to be higher than with conventional tubular plates. We hypothesize that the benefits of locking plates in terms of biomechanical properties are outweighed by a higher risk of wound complications.

**Methods:**

We conducted a retrospective review of fibula fractures treated by osteosynthesis with either a conventional one-third tubular plate or an anatomically shaped locking plate from January 1, 2015 to December 31, 2021. We recorded baseline data and relevant comorbidities and defined the need for revision surgery due to wound-related or mechanical complications as primary endpoints.

**Results:**

A total of 595 out of 727 patients were eligible for our study. Of these 595 fractures, 526 were fixed with a one-third tubular plate, 69 with a locking plate. Revision surgery was required in 54 patients, in 51 cases due to wound complications. Three patients required revision surgery for mechanical reasons and all of them were younger than 40 years of age, have not been diagnosed with osteoporosis, but experienced complex fracture types. As the third tubular plate and locking plate groups differed in terms of age and comorbidities, we performed a 2:1 matching based on age and gender, leaving data from 138 patients receiving a third tubular plate. While the two groups were comparable in many aspects, the rate of wound complications was significantly higher in the locking plate group. Although the locking plate group had a higher percentage of diabetes mellitus, there was no correlation between this comorbidity and the higher revision rate in this group.

**Conclusions:**

Our data do not support the general use of locking plates in the treatment of distal fibular fractures. The risk of mechanical complications in osteoporotic ankle fractures seems to be overrated, as there were no mechanical revisions in the osteoporotic subgroup. The rate of wound-related revision surgery was significantly higher after the use of locking plates. This might be attributed to the greater thickness of locking plates.

## Background

**Fig. 1:**
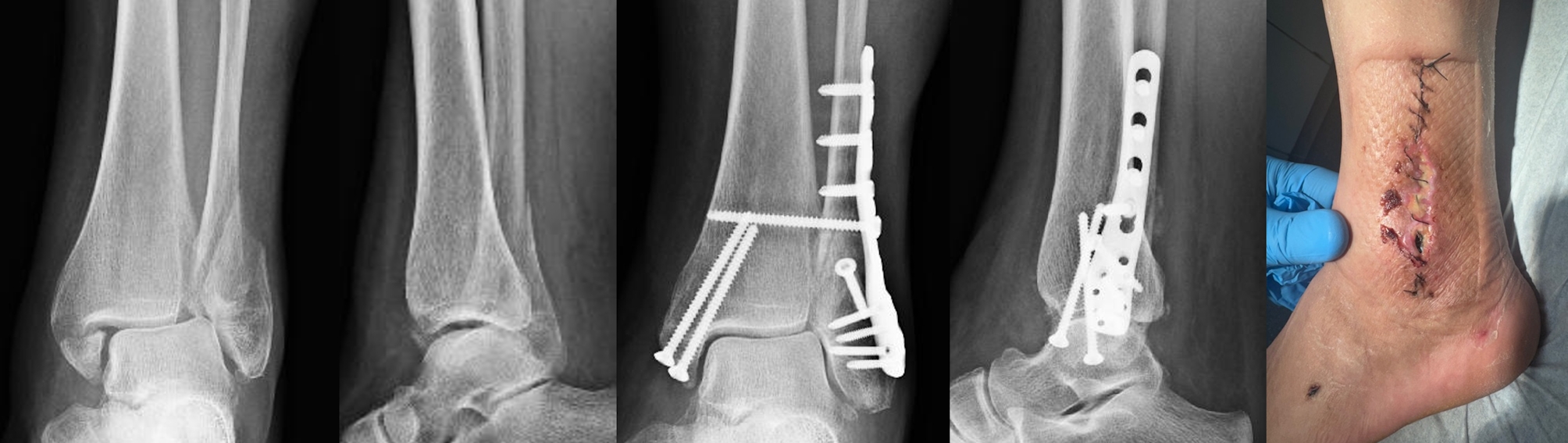
Case of a 57-year-old patient with a history of alcohol abuse, malnutrition and chronic hepatitis, who developed an early wound infection following fixation of a distal fibula fracture with an anatomic locking plate

In the past two decades, locking plates are increasingly used for the treatment of distal fibula fractures [1] and several biomechanical studies indicate an advantage of locking plates compared to the conventional one-third tubular plate, particularly in osteoporotic bone [2, 3]. However, it remains to be elucidated whether the advantageous biomechanical features of the locking plate translate into an improvement of the outcome. A recent systematic review and metaanalysis of 18 studies (4243 fractures) found no difference in the functional outcome and the complication rate after use of a locking or a non-locking plate [4]. Some studies report a higher rate of wound complications when using locking plates, which is attributed to the greater plate thickness and the lateral position of the plates examined [5, 6] (Fig. 1).

Against this background, we hypothesize that the benefits of locking plates in terms of the putative better biomechanical properties are outweighed by a greater risk of wound complications. To improve patient safety, we need to obtain better clinical data on the advantages and disadvantages of locking plates.

Therefore, we conducted a single-center chart review paying particular attention to the occurrence of wound and mechanical complications in patients undergoing distal fibular plating with locking plates and non-locking plates, respectively.

## Methods

We hypothesized that locking plates are not superior to non-locking plates in regards to mechanical and wound complications. To test this hypothesis, we conducted a single-center retrospective chart review at a tertiary university medical center. Our clinical information system was searched to retrieve files from all patients receiving internal fixation of a lower leg fracture (ICD-10 S82) in combination with treatment by internal fixation with a standard (non-locking) one-third tubular plate or an anatomical locking plate (OPS 5-793.3R, 5-793.KR, 5-794.2R, 5-794.KR) between January 1, 2015 and December 31, 2021. All records were pseudonymized and this retrospective analysis was approved by the local ethics committee (approval number A 2024-0049). Apart from baseline anthropometric characteristics (age at time of fracture, sex), the presence of diabetes, arterial hypertension, osteoporosis, peripheral artery disease as well as smoking behavior and alcohol abuse at the time of internal fixation were gathered by chart review. Revision surgery due to either wound or mechanical complications within one year after internal fixation were considered as primary endpoint, whereas duration of surgery (cut-seam interval) served as a secondary measure of outcome. Wound-related complications were identified according to the criteria of Metsemakers et al. [7] whereas failure of fixation, implant loosening or breakage, or non-union were summarized as mechanical complications.

Patients with a fibula fracture unrelated to a malleolar fracture, i.e., one of the AO/OTA regions 42 and 43 were excluded. Similarly, those receiving an antibacterially coated locking plate or being transferred from another hospital for revision surgery were not eligible.

**Fig. 2:**
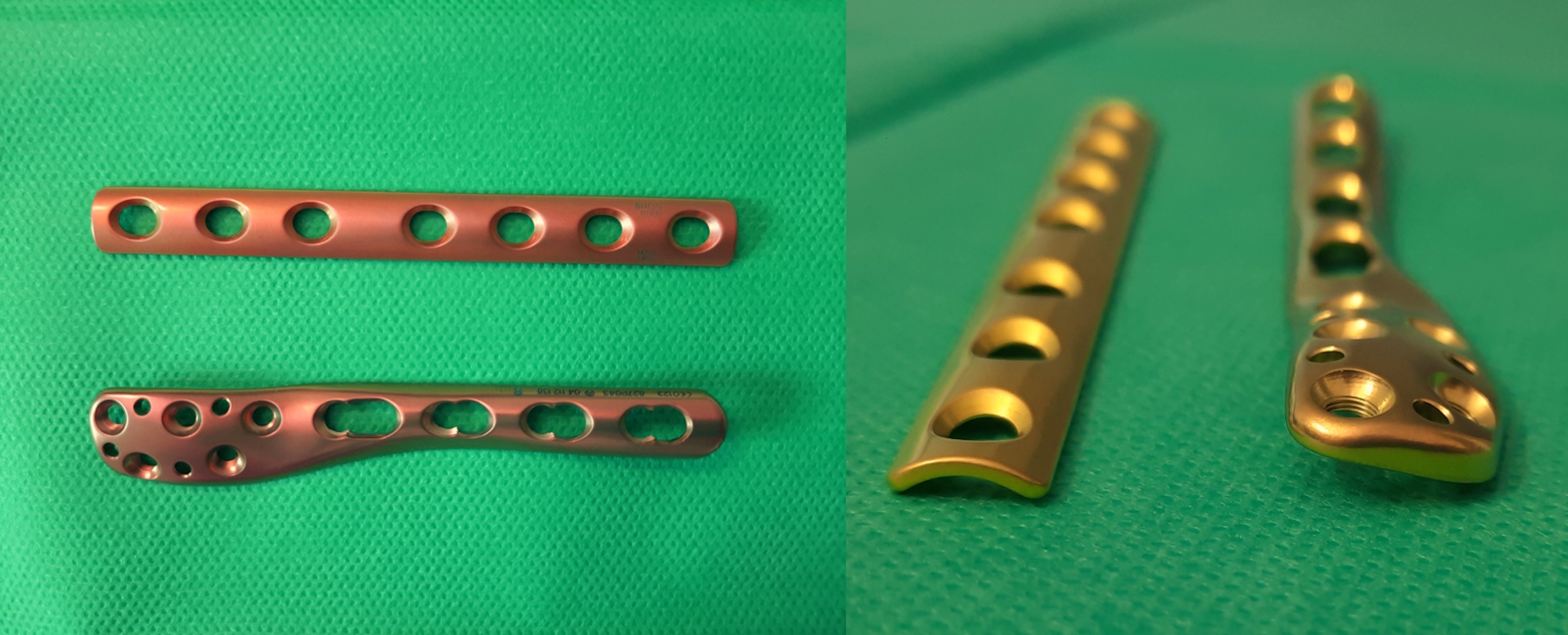
Plates used throughout this study for treatment of distal fibular fractures (**A**) Top view of the 3.5 mm one-third tubular plate (DePuy Synthes) (top) and the LCP Lateral Distal Fibula Plate (DePuy Synthes) (bottom). (**B**) Frontal view of the 3.5 mm one-third tubular plate (DePuy Synthes) (left) and the LCP Lateral Distal Fibula Plate (DePuy Synthes) (right)

An anatomically shaped locking plate for the lateral aspect of the distal fibula with combi-holes in the proximal segment and holes for multidirectionally angular stable fixation in the distal segment (2.7 mm/3.5 mm LCP Lateral Distal Fibula Plat, Johnson & Johnson Medical, Depuy Synthes, Konrad-Zuse-Strasse 19, 66459 Kirkel, Germany) and a 3.5 mm one-third tubular plate (2.7 mm/3.5 mm LCP Lateral Distal Fibula Plate, Johnson & Johnson Medical, Depuy Synthes, Konrad-Zuse-Strasse 19, 66459 Kirkel, Germany) were regularly available at our institution (Fig. 2). The decision for a particular system was left to the discretion of the surgeon although the manufacturer’s recommendations were followed in case of osteopenic bone.

All operations were performed by specialized orthopedic surgeons themselves or under their supervision. Preoperative preparation ensured, that the extent of soft tissue swelling allowed for definitive fracture repair. Clinical signs of the reduction in swelling included the absence of new tension blisters, the appearance of small skin folds, the so-called wrinkle sign, and a decrease in circumference. The prep routinely used in the study period was BD ChloraPrep™ (Becton Dickinson, Heidelberg, Germany). Either the direct lateral approach or the posterolateral approach was chosen. In all cases, an open reduction and lateral plate osteosynthesis was performed using either a conventional one-third tubular plate or an anatomically shaped locking plate in a lateral position. If the syndesmosis complex was unstable, a syndesmotic screw was used. As our study criteria excluded Maisonneuve fractures, this applied to selected fractures of AO types 44-B and 44-C1. To determine or exclude instability of the syndesmosis, the stability of the syndesmosis complex was routinely tested intraoperatively after stabilization of all fractures by posterolateral traction on the proximal fibula with a bone hook. The layer-by-layer wound closure including skin suturing was performed by the surgeon himself.

### Statistical analysis

All information was summarized in a spreadsheet (Microsoft^®^ Excel Version 16. 0.5422.1000, Microsoft Corporation, USA) and the mode of osteosynthesis, i.e., non-locking plate or anatomical locking plate, was used for categorization while the need for revision surgery and the duration of the index surgery served as primary and secondary endpoints. Assumptions of normality were tested by means of the Shapiro-Wilk test and evaluated graphically using histograms and Q-Q-plots. That data was described as mean and standard deviation or median and interquartile range (IQR), as appropriate. Analysis of variance was used to compare the metric characteristics. With respect to the primary outcome the Student’s t-test and Welch’s t-test were applied to check for significant differences between groups with normally distributed data and similar variance respectively dissimilar variance, whereas Mann-Whitney U test was performed for non-normally distributed data. Categorical variables are presented as percentages and the Chi-square test was applied for comparison between groups. A two-tailed p value ≤ 0.05 was considered statistically significant. Analysis was performed using SPSS version 27 (SPSS GmbH, Germany) and Sigma Plot 13 (Systat Software GmbH, Germany) were used for visualization of results.

As it is still a matter of discussion, whether and which patients at all benefit from locking vs. non-locking plates in terms of clinical outcome, all patients being at least 15 years of age at time of fracture were initially included. However, and as to be expected locking plate osteosynthesis was preferentially used in the elderly, i.e. a subgroup with a high incidence of comorbidities. In addition, the absolute number of patients treated with locking plates is significantly lower than the number of patients treated with conventional plates. To account for this apparent dysbalance between patients treated with locking and non-locking osteosynthesis, we performed a 2:1 matching for the parameters age and gender.

## Results

**Fig. 3:**
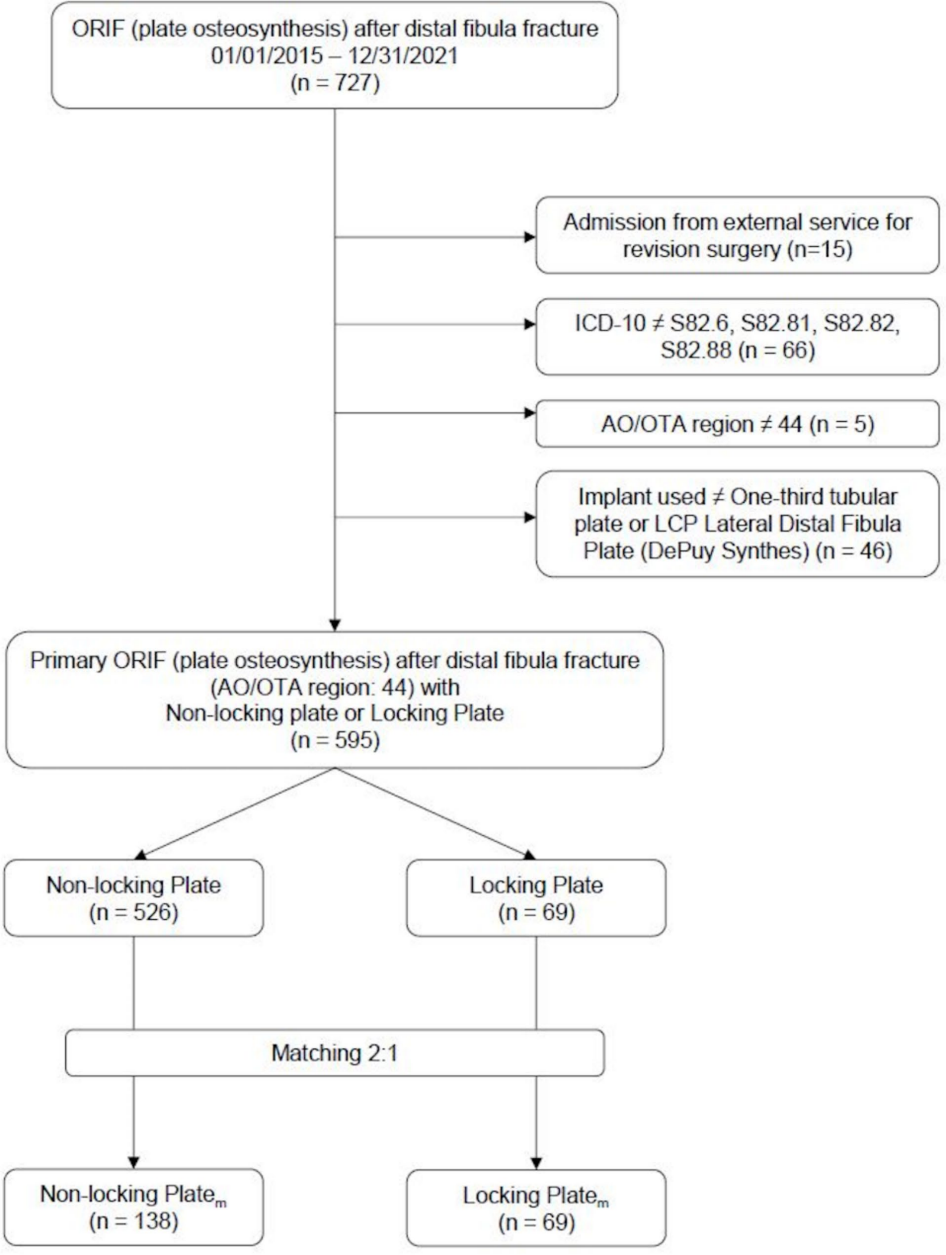
Flow-chart demonstrating the identification of the study population Files were retrieved from the clinical information system and checked for matching to the inclusion criteria. For each individual of the locking plate group two patients of the third-tubular plate group were matched for age and sex

Out of 727 cases 132 did not meet our inclusion criteria and were consequently excluded from the analysis (Fig. 3). Thus, the records of 595 patients (238 males) were considered for analysis and a total of 54 patients underwent revision surgery. 51 out of 54 revision procedures (94.4%), were due to infectious complications. Mechanical complications were the cause of revision surgery in three patients, who were under 40 years of age and had suffered complex injuries (one bimalleolar fracture, two trimalleolar fractures) without signs of osteoporosis. 69 patients (17 m) received an anatomical locking plate while in 526 patients (221 m) a conventional one-third tubular plate was used. These two groups differ significantly in terms of age and sex as well as with respect to the presence of arterial hypertension, diabetes mellitus, and osteoporosis (Table 1).

**Table 1:** Overall

		Overall (*n* = 595)	Third tubular plate (*n* = 526)	Locking plate (*n* = 69)	*p*-value
Age [years], mean ± std		54.39 ± 18.69	52.16 ± 18.21	71.41 ± 12.59	**< 0.001** ^a^
Sex, n (%)	m f	238 (40.0) 357 (60.0)	221 (42.0) 305 (58.0)	17 (24.6) 52 (75.4)	**0.006** ^b^
BMI [kg/m²], median (max; min)		26.90 (55.9;16.2)	26.90 (55.9; 16.2)	27.04 (43.3; 18.0)	0.355^c^
AO/OTA, n (%)	44 A 44B 44 C	25 (4.2) 469 (78.8) 101 (17.0)	23 (4.4) 413 (78.5) 90 (17.1)	2 (2.9) 56 (81.2) 11 (15.9)	0.811^b^
Type of ankle fracture, n (%)	unimalleolar bimalleolar trimalleolar	277 (46.6) 110 (18.5) 208 (35.0)	257 (48.9) 92 (17.5) 177 (33.7)	20 (29.0) 18 (26.1) 31 (44.9)	**0.007** ^b^
Open fracture, n (%)		16 (2.7)	11 (2.1)	5 (7.2)	**0.029** ^b^
ASA-Score, mean ± std	1 2 3 4	117 (21.8) 312 (58.1) 105 (19.6) 3 (0.6)	115 (24.1) 279 (58.5) 80 (16.8) 3 (0.6)	2 (3.3) 33 (55.0) 25 (41.7) 0 (0.0)	**< 0.001** ^b^
External fixator, n (%)		84 (14.1)	68 (12.9)	16 (23.2)	**0.021** ^b^
Comorbidity, n (%)	Diabetes mellitus Peripheral Artery Disease Smoking Arterial hypertension Osteoporosis Alcohol abuse	78 (13.1) 13 (2.2) 152 (25.5) 262 (44.0) 62 (10.4) 43 (7.2)	52 (9.9) 11 (2.1) 140 (26.6) 210 (39.9) 40 (7.6) 37 (7.0)	26 (37.7) 2 (2.9) 12 (17.4) 52 (75.4) 22 (31.9) 6 (8.7)	**< 0.001** ^b^ 0.655^b^ 0.099^b^ **< 0.001** ^b^ **< 0.001** ^b^ 0.620^b^
Time to definitive surgery [days], median (max; min)		6 (49; 0)	6 (49; 0)	4 (26; 0)	**< 0.001** ^c^
Day of the week, n (%)	Mo Tu We Th Fr Sa Su	128 (21.5) 86 (14.5) 111 (18.7) 82 (13.8) 147 (24.7) 23 (3.9) 18 (3.0)	111 (21.1) 73 (13.9) 103 (19.6) 72 (13.7) 131 (24.9) 19 (3.6) 17 (3.2)	17 (24.6) 13 (18.8) 8 (11.6) 10 (14.5) 16 (23.2) 4 (5.8) 1 (1.4)	0.547^b^
Time of surgery, n (%)	8 am − 4 pm 4 pm − 0 pm 0 am − 8 am	420 (70.6) 167 (28.1) 8 (1.3)	382 (72.6) 136 (25.9) 8 (1.5)	38 (55.1) 31 (44.9) 0 (0.0)	**0.003** ^b^
Duration of surgery [min], median (max; min)		74.00 (450; 18)	71 (450; 18)	95 (196; 37)	**< 0.001** ^c^
Drain, n (%)		419 (70.4)	366 (69.6)	53 (76.8)	0.216^b^
Syndesmotic screw, n (%)		270 (45.4)	231 (43.9)	39 (56.5)	**0.048** ^b^
Revision surgery, n (%)		54 (9.1)	39 (7.4)	15 (21.7)	**< 0.001** ^b^
Time of revision surgery	Early (< 4 postoperative weeks) Late (> 4 postoperative weeks)	15 (29,4) 36 (70,6)	11 (30,6) 25 (69,4)	4 (26.7) 11 (73.3)	1^b^

^a^ Welch’s t-test

^b^ Chi-squared test

^c^ Mann–Whitney U test

Both groups were quite comparable in terms of the frequency of open fractures, the temporary use of an external fixator and the use of wound drains. In the locking plate group, syndesmotic screws were used with significantly higher frequency and duration of surgery was significantly longer compared to the non-locking plate group.

On the one hand, the incidence of diabetes mellitus and osteoporosis increases with age, and on the other hand, it is known that one of these concomitant diseases has a negative effect on wound healing. We therefore applied age and gender as criteria for matching of non-locking plate to locking plate patients (ratio of 2:1) (Table 2). With the exception of diabetes mellitus (27 out of 138 non-locking plate patients vs. 26 out of 69 locking plate patients, *p* ≤ 0.005) the distribution of the aforementioned comorbidities in the matched non-locking plate group was similar to that of the locking plate group. The median Body Mass Index (BMI) also showed no significant difference between both groups. Further, it was found that revision surgery due to wound complications was not related to the presence of diabetes mellitus or a high BMI. (Table 3).

**Table 2:** Matched pairs 2:1

		Matched Pairs (*n* = 207)	Third tubular plate_m_ (*n* = 138)	Locking plate_m_ (*n* = 69)	*p*-value
Age [years], mean ± std		70.47 ± 12.08	70.01 ± 11.84	71.41 ± 12.59	0.434^a^
Sex, n (%)	m f	51 (24.6) 156 (75.4)	34 (24.6) 104 (75.4)	17 (24.6) 52 (75.4)	1^b^
BMI [kg/m²], median (max; min)		27.10 (55.9; 18.0)	27.20 (55.9; 19.2)	27.04 (43.3; 18.0)	0.879^c^
AO/OTA, n (%)	44 A 44B 44 C	8 (3.9) 164 (79.2) 35 (16.9)	6 (4.3) 108 (78.3) 24 (17.4)	2 (2.9) 56 (81.2) 11 (15.9)	0.837^b^
Type of ankle fracture, n (%)	unimalleolar bimalleolar trimalleolar	87 (42,0) 44 (21,3) 76 (36,7)	67 (48,6) 26 (18,8) 45 (32,6)	20 (29,0) 18 (26,1) 31 (44,9)	**0.027** ^b^
Open fracture, n (%)		9 (4.3)	4 (2.9)	5 (7.2)	0.164^b^
ASA-Score, mean ± std	1 2 3 4	14 (7.6) 99 (53.8) 69 (37.5) 2 (1.1)	12 (9.7) 66 (53.2) 44 (35.5) 2 (1.6)	2 (3.3) 33 (55.0) 25 (41.7) 0 (0)	0.315^b^
External fixator, n (%)		34 (16.4)	18 (13.0)	16 (23.2)	0.063^b^
Comorbidity, n (%)	Diabetes mellitus Peripheral Artery Disease Smoking Arterial hypertension Osteoporosis Alcohol abuse	53 (25.6) 8 (3.9) 33 (15.9) 147 (71.0) 52 (25.1) 14 (6.8)	27 (19.6) 6 (4.3) 21 (15.2) 95 (68.8) 30 (21.7) 8 (5.8)	26 (37.7) 2 (2.9) 12 (17.4) 52 (75.4) 22 (31.9) 6 (8.7)	**0.005** ^b^ 0.721^b^ 0.687^b^ 0.330^b^ 0.113^b^ 0.558^b^
Time to definitive surgery [days], median (max; min)		5 (26; 0)	5 (17; 0)	4 (26; 0)	**0.004** ^c^
Day of the week, n (%)	Mo Tu We Th Fr Sa Su	49 (23.7) 30 (14.5) 44 (21.3) 20 (9.7) 48 (23.2) 9 (4.3) 7 (3.4)	32 (23.2) 17 (12.3) 36 (26.1) 10 (7.2) 32 (23.2) 5 (3.6) 6 (4.3)	17 (24.6) 13 (18.8) 8 (11.6) 10 (14.5) 16 (23.2) 4 (5.8) 1 (1.4)	0.121^b^
Time of surgery, n (%)	8 am − 4 pm 4 pm − 0 pm 0 am − 8 am	131 (63.3) 73 (35.3) 3 (2.2)	93 (67.4) 42 (30.4) 2 (1.4)	38 (55.1) 31 (44.9) 0 (0.0)	0.069^c^
Duration of surgery [min], median (max; min)		78.00 (218; 25)	70 (218; 25)	95 (196; 37)	**< 0.001** ^a^
Drain, n (%)		147 (71.0)	94 (68.1)	53 (76.8)	0.194^b^
Syndesmotic screw, n (%)		96 (46.4)	57 (41.3)	39 (56.5)	**0.038** ^b^
Revision surgery, n (%)		30 (14.5)	15 (10.9)	15 (21.7)	**0.036** ^b^
Time of revision surgery	Early (< 4 postoperative weeks) Late (> 4 postoperative weeks)	6 (20.0) 24 (80.0)	2 (13.3) 13 (86.7)	4 (26.7) 11 (73.3)	0.651^b^

^a^ Student’s t-test

^b^ Chi-squared test

^c^ Mann–Whitney U test

**Table 3:** Subgroup analysis, wound complications requiring revision surgery

		Revisions (*n* = 30)	Third tubular plate_*r*_ (*n* = 15)	Locking plate_*r*_ (*n* = 15)	*p*-value
Diabetes mellitus, n (%)		13 (43.3)	5 (33.3)	8 (53.3)	0.269^a^
BMI [kg/m²], median (max; min)		26.62 (47.8; 18.0)	27.00 (47.8; 22.0)	26.24 (33.7; 18.0)	0,367^b^
Obesity, n (%)	Under/normal weight Overweight Class 1 obesity Class 2 obesity Class 3 (high-risk) obesity	12 (40.0) 10 (33.3) 6 (20.0) 1 (3.3) 1 (3.3)	6 (40.,0) 3 (20.0) 4 (26.7) 1 (6.7) 1 (6.7)	6 (40.0) 7 (46.7) 2 (13.3) 0 (0) 0 (0)	0,371^a^
Type of ankle fracture, n (%)	unimalleolar bimalleolar trimalleolar	9 (30.0) 8 (26.7) 13 (43.3)	5 (33.3) 4 (26.7) 6 (40.0)	4 (26.7) 4 (26.7) 7 (46.7)	0.910
Time to definitive surgery [days], median (max; min)		4.00 (37; 0)	4.00 (11; 0)	3.00 (7; 0)	0.379^b^
Duration of surgery [min], median (max; min)		100.00 (218; 11)	77.00 (218; 46)	118.00 (196; 37)	0.350^b^
Syndesmotic screw, n (%)		17 (56.7)	6 (40.0)	11 (73.3)	0.065^a^

^a^ Chi-squared test

^b^ Mann–Whitney U test

## Discussion

This retrospective chart review clearly showed that the vast majority of all revision procedures were due to wound complications and not due to mechanical complications. The latter were only found in three young and non-osteoporotic patients from the group of patients with non-locking plates, while they did not occur at all in patients with clinically diagnosed osteoporosis. This stands in sharp contrast to the general assumption that mechanical complications are preferentially seen in osteoporotic bone [8, 9].

This finding is of particular importance as, similar to other authors [5, 6], we have found a higher rate of wound complications following the fixation with locking plates. Apparently, the biomechanical superiority of locking plates does not come into play clinically, as stabilization with non-locking plates also appears to guarantee sufficient safety against mechanical complications. While we have not conducted regular follow-up to determine patient-related outcomes, it is known from previous studies that the use of locking plates provides little functional benefit [10–14].

Like other authors [6], we attribute the higher rate of wound complications to the greater plate thickness. Another important factor is the position of the implant. Wenger and co-workers [15] found a higher rate of reoperation following lateral than after posterolateral plate positioning. In this study, we used a first-generation locking plate anatomically shaped for lateral positioning. Considering the higher complication rates found in this study and in the literature, the use of this type of implant could be disadvantageous for patients with an increased risk of wound complications. Based on these findings, various manufacturers have now developed locking plates with a reduced plate thickness. In addition, anatomically shaped plates have been introduced for alternative positioning (mainly posterior and posterolateral). Future studies will show whether these changes will lead to a lower tendency to wound complications.

However, the key finding in recent years seems to be that the priority in the treatment of ankle fractures, particularly in high-risk patients, should be soft tissue management rather than fixation with maximum stability. In this context, it should also be emphasized that too little use is made of the option of conservative treatment for stable fractures [16, 17]. For unstable fractures, the use of retrograde fibula locking nails or simple retrograde screws with or without additional external fixation are promising alternatives to plate fixations [18, 19]. A systematic review and metaanalysis found superior clinical outcomes and lower complication rates when using locked intramedullary nails compared to plate fixation [20].

### Limitations of the study

As this was a retrospective study, the choice of treatment was based on the individual surgeon’s clinical judgment. However, the recommendations of the manufacturer were considered and thus locking plates were preferentially chosen in elderly patients with impaired bone quality. Our analysis is based on the information available via chart review and no follow-up measures were applied. Furthermore, we did not consider the effects of an improved plate design, e.g., thickness and/or positioning.

## Conclusions

Our data question the current notion, that mechanical complications occur preferentially in osteoporotic fractures. While older patients are thought to benefit from stable fixation with a locking plate, this is contrasted by a non-negligible rate of wound complications.

## Data Availability

The dataset analyzed during the current study is available from the corresponding author on reasonable request.

## Abbreviations

AO/OTA: Arbeitsgemeinschaft fuer Osteosynthesefragen/Orthopaedic Trauma Association
ASA: American Society of Anesthesiologists
BMI: Body Mass Index
ICD-10: International Statistical Classification of Diseases and Related Health Problems, Version 10
LCP: Locking Compression Plate
OPS: Operationen- und Prozedurenschluessel, German Modification of the International Classification of Procedures in Medicine (ICPM)
